# Phytotoxin coronatine enhances heat tolerance via maintaining photosynthetic performance in wheat based on Electrophoresis and TOF-MS analysis

**DOI:** 10.1038/srep13870

**Published:** 2015-09-08

**Authors:** Yuyi Zhou, Mingcai Zhang, Jianmin Li, Zhaohu Li, Xiaoli Tian, Liusheng Duan

**Affiliations:** 1 State Key Laboratory of Plant Physiology and Biochemistry, Engineering Research Center of Plant Growth Regulator, Ministry of Education, College of Agronomy and Biotechnology, China Agricultural University, No 2 Yuanmingyuan Xi Lu, Haidian District, Beijing 100193, China

## Abstract

Coronatine (COR) is a phytotoxin produced by *Pseudomonas syringae*. Its structure is similar to Jasmonates, which play a number of diverse roles in plant defense. Both have the COI1 plant receptor, so coronatine can manipulate plant hormone signaling to access nutrients and counteract defense responses. In addition to the hormone system, coronatine affects plant nitrogenous metabolism and chloroplast ultrastructure. In this study, we first examined a typical nitrogen-losing phenotype, and used the polyacrylamide gel approach to demonstrate soluble total protein patterns in a time-course experiment under different temperature conditions. We then employed dimensional gel electrophoresis technology (2-DE) and MALDI-TOF-MS to sequester and identify the sensitive proteins. We found a total of 27 coronatine sensitive proteins, 22 of which were located in the chloroplast and 6 of which were directly involved in photosynthesis. Finally, we measured levels of chlorophyll and photosynthetic performance to reveal the phenotypic effect of these proteins. Taken together, these results demonstrated that coronatine enhanced heat tolerance by regulating nitrogenous metabolism and chloroplast ultrastructure to maintain photosynthetic performance and reduce yield loss under heat stress.

Wheat (*Triticum aestivum L*.) is usually exposed to high temperature during grain filling. Plant stress during this stage directly affects grain numbers and grain mass. It’s classified as one of the major adversities for wheat^1^,^2^,^3^, this risk will increase because of the changing climate^4^. Heat stress is a significant factor restricting plant growth, it regulates multiple processes in gene expression in order to globally repress protein synthesis and selectively up regulate stress response proteins^5^,^6^,^7^,^8^,^9^. Photosynthesis is the most sensitive physiological process affected by heat stress in plants^10^, so reduction of photosynthesis leads to a reduction in growth and grain yield^11^,^12^.

Coronatine is a chlorosis-inducing non-host-specific phytotoxin produced by several members of the *Pseudomonas syringae* pathovars^13^. It is a structural mimic of jasmonates, but more active than jasmonates in some functions, in plants, both share the same receptor, coronatine insensitive1 (COI1)^14^,^15^, so coronatine can manipulate plant hormone signaling to access nutrients and counteract defense responses^16^,^17^,^18^. Coronatine in a high concentration (normally more than 10 μM) can lead to leaf chlorosis, anthocyanin production, ethylene emission, auxin synthesis^19^,^20^, opening of stomata for bacterial entry, bacterial growth in the apoplast, systemic susceptibility, and disease symptoms^21^,^22^. It also increases the accumulation of defense-related protease inhibitors and secondary metabolites, such as volatiles, nicotine and alkaloid^23^. Low coronatine amounts (normally less than 1 μM) plays an important role in resisting abiotic stress, such as improving salt stress tolerance in cotton^24^, cold resistance in cucumber^25^, drought tolerance in rice^26^, maize^27^, cauliflower^28^ and soybean^29^, and heat stress in chickpea^30^. Most coronatine researches have focused on explaining the mechanism of coronatine in hormone pathways^31^,^32^. However, our study demonstrates new insights on the mechanism of coronatine enhancing heat tolerance.

## Results

### Coronatine enhanced heat tolerance in wheat, resulting in leaves showing greener phenotype and high relative leaf water content.

**Figure 1:**
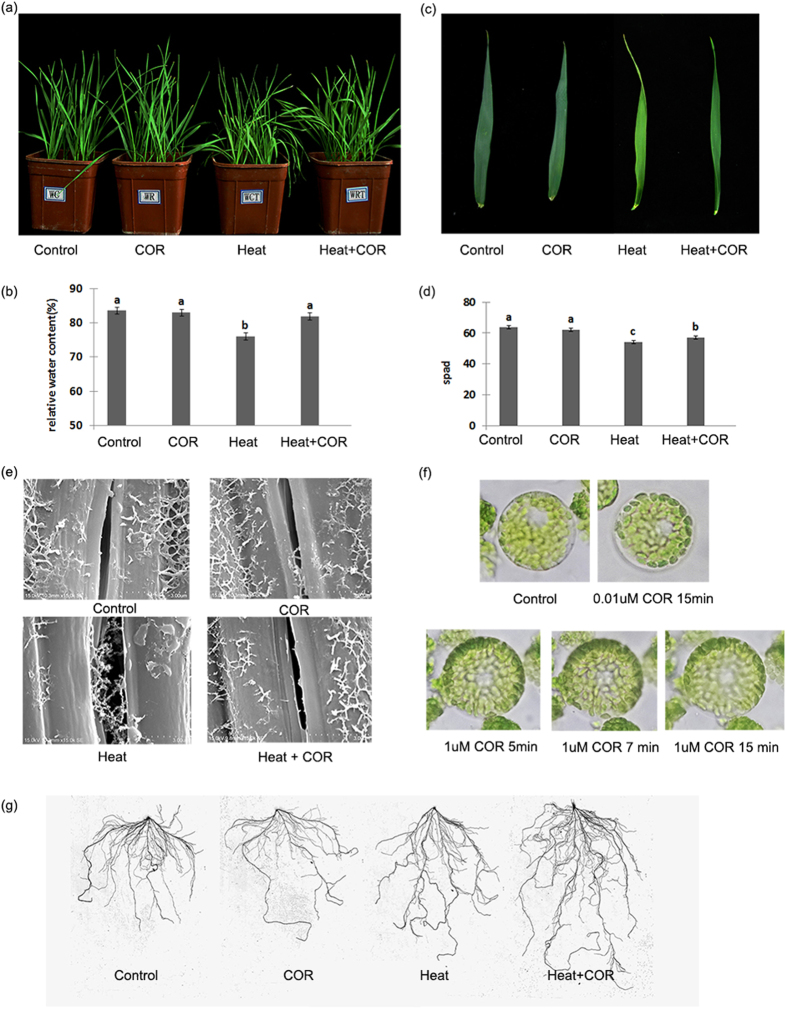
Coronatine induced physiological phenotype in wheat under heat stress. (**a**) Phenotype of wheat leaves in different treatment. Four treatments: Control: 20 °C for 48 hrs after water pretreatment for 24 hrs; COR: 20 °C for 48 hrs after coronatine pretreatment for 24 hrs; Heat: 40 °C treatment for 48 hrs after water pretreatment for 24 hrs; COR + Heat: 40 °C for 48 hrs after coronatine pretreatment under 20 °C for 24 hrs. (**b**) Relative leaf water content of wheat leaves under different treatments. The relative water content ratio is shown as mean ± SD from 6 replicates (n = 15 lines/replicate; Bars labeled with different letter are significantly different at P > 0.05 as determined by LSD test) corresponding with figure a phenotype. (**c**) Phenotype of wheat flag leaves in different treatment. (**d**) Chlorophyll level of flag leaves under different treatments. The SPAD value indicating chlorophyll level in the flag leaves are shown as mean ± SD from 6 replicates (n = 15 lines/replicate; Bars labeled with different letter are significantly different at P > 0.05 as determined by LSD test) corresponding with figure c phenotype. (**e**) Stomata of wheat seedlings under different treatment in 15.0 kV 10.3 mm × 15.0 k Secondary Electron. (**f**) Protoplast of wheat second leaf under different concentration coroantine treatment. (**g**) Root phenotype of wheat seedlings under different treatment.

Coronatine preserved the green phenotype of wheat leaves under heat stress (Fig. 1c). The SPAD value indicates chlorophyll level in the flag leaves, coronatine pretreated leaves were 7.89% higher than control plants under heat stress (Fig. 1d). Meanwhile, the protoplast result also showed this phenomenon in cell level, low intercellular coronatine (0.01 μM) could make the chloroplast much greener, while high intercellular coronatine (1 μM) may destroy the cell membrane (Fig. 1f). Additionally, coronatine maintained high relative leaf water content under heat stress conditions (Fig. 1a,b), and reduced the damage of stomata from heat. The stomata under heat stress spited out some compounds and the cell wall/membrane was not integrated (Fig. 1e). Coronatine also stimulated root growth, enhancing uptake of water and nutrition under heat stress (Fig. 1g, Table 1).

### Coronatine inducible proteins under heat stress in wheat leaves

**Figure 2:**
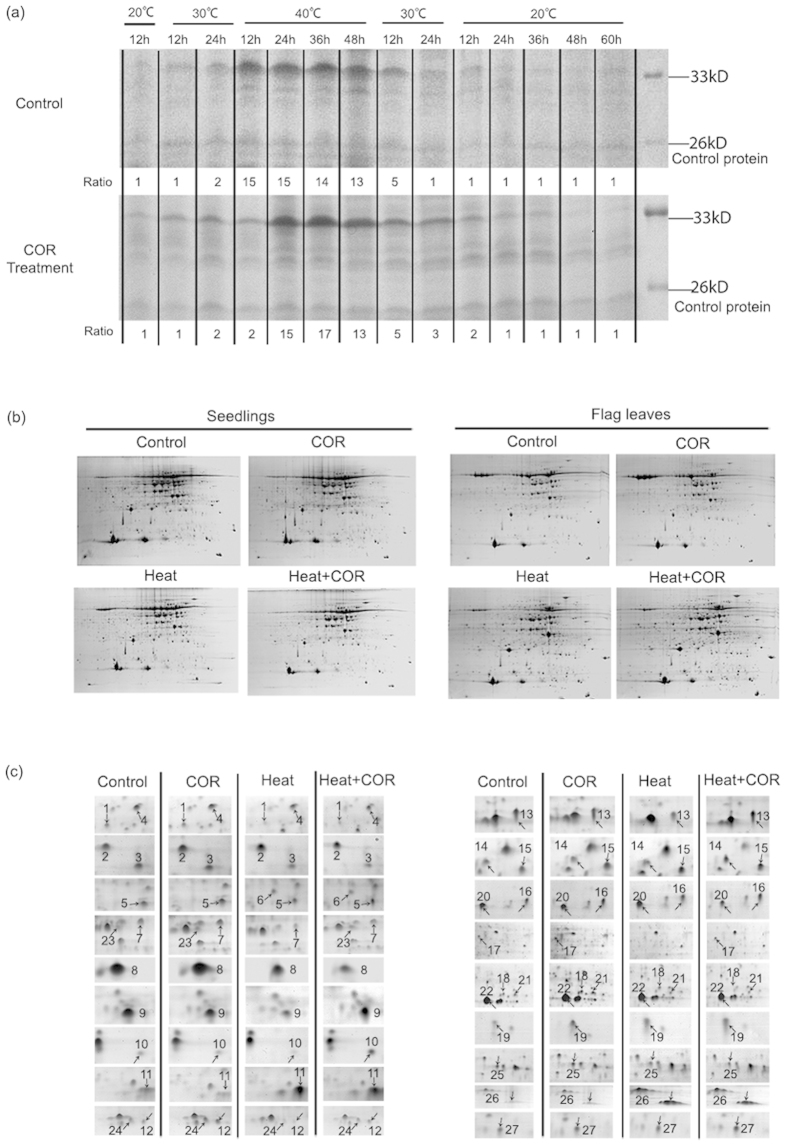
Protein pattern induced by coronatine. (**a**) Time course of soluble protein patterns according to temperature changes under different treatment. Proteins in 26 kD are stable as control protein; proteins in 37 kD are a group of heat shock proteins. (**b**) Representative 2-DE gels of leaf tissues under different treatment. (**c**) The observed variation pattern in protein spot. Numbers of spots indicated by the arrows refer to the Table 2.

**Figure 3:**
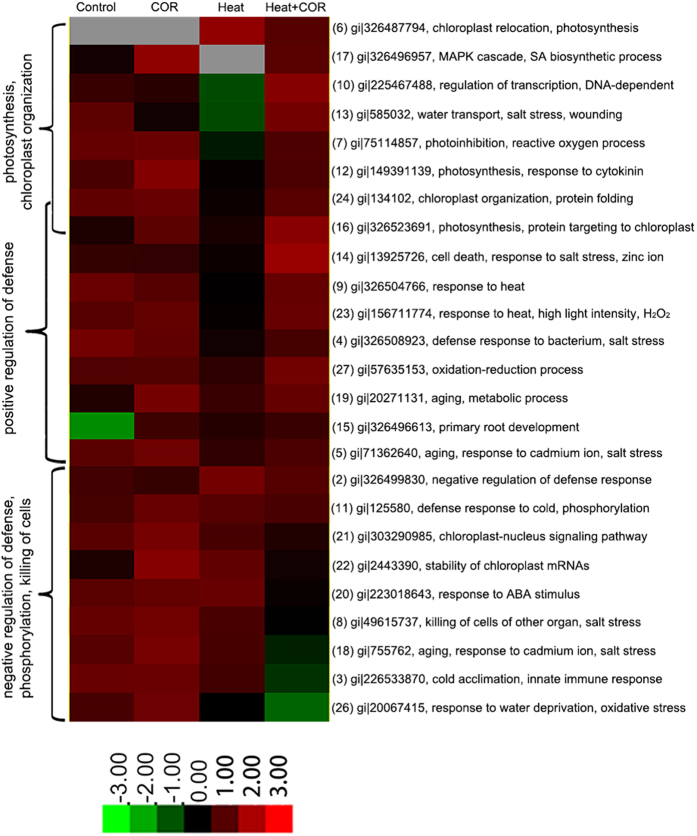
Biological process analysis of proteins induced by coronatine under heat stress. Expression diagram of selected proteins that are differentially regulated by treatment Control, COR, Heat and Heat + COR. Each protein was classified with respect to its cellular component, biological process, and molecular function. Three major groups are indicated: group I, proteins belonging to photosynthesis and chloroplast organization; group II, proteins involved in positive regulation of defense; group III, proteins responsive to negative regulation of stress, phosphorylation and plant immune system. Protein expression pattern analysis was performed using Cluster 3.0.

We performed time course experiment to monitor production of soluble total protein. Results showed that coronatine pretreatment could change protein expression patterns. Heat shock proteins were induced by coronatine at an early stage, and disappeared at a later stage. Heat shock proteins reached a maximum concentration at 36 hours after coronatine treatment, and 24 hours delay than control plants (Fig. 2a). Depend on the time course results, we employed dimensional gel electrophoresis technology to identify twenty seven coronatine inducible proteins. Twenty two of these proteins are located in the chloroplast, and six are directly involved in photosynthesis (Fig. 2b,c; Table 2). When heat stress occurred, most of proteins significantly decreased their expression level, while coronatine pretreatment increased the stability of these proteins under heat stress (Figs 2c and 3).

Coronatine inducible proteins regulated nitrogenous metabolism and protein process. Glutamine synthetase (GS) plays an essential role in the metabolism of nitrogen (N) by catalyzing the condensation of glutamate and ammonia to form. GS2 (gi|71362640) is located mainly in the chloroplasts and mitochondria, and involved in nitrite reduction and photorespiration. Coronatine pretreatment increased GS2 expression level under heat stress. Cysteine synthase (gi|585032) and protein disulfide isomerase 2 (gi|13925726) are important in protein processes and structure rebuilding. Coronatine pretreatment significantly increased their expression level under heat stress (Figs 2c and 3).

Coronatine inducible proteins regulated chloroplast ultrastructure and photosynthesis. We found twenty two coronatine sensitive proteins located in the chloroplast, distributed in the chloroplast envelope, chloroplast stroma and chloroplast thylakoid membrane. 30S ribosomal protein S1 (gi|149391139) is involved in the chlorophyll biosynthetic process. OsFTSH2 (gi|75114857) plays a key role in PSII associated light-harvesting complex II catabolic process. CPN-60 alpha (gi|134102) is crucial to chloroplast organization and protein folding. Predicted protein (gi|326523691) is a target of chloroplast and directly involved in photosynthesis. When heat stress occurred, these proteins were unstable and degraded, while coronatine pretreatment increased their stability, and then increased the stability of chloroplast ultrastructure and membrane to maintain a higher photosynthesis under heat stress. (Table 2; Figs 2c and 3).

Coronatine inducible proteins also regulated plant defense and innate immune response. Eighteen of coronatine inducible proteins were response to abiotic stress, and eleven of them were positive regulation of heat stress. Coronatine pretreatment significantly increased the expression level of these eleven proteins under heat stress. Predicted protein (gi|326499830), a negative regulator of defense response, and pathogenesis-related protein 4 (gi|49615737), involved in killing cells of other organs, coronatine pretreatment decreased their expression level under heat stress. There were also several coronatine inducible proteins whose variation looked novel to stress: GST, cp31BHv and Ps16 are innate immune response proteins. Coronatine pretreatment decreased their expression level under heat stress. This result reflected the property of phytotoxin coronatine, it enhanced plant defense in some way, and stimulated plant immune system at the same time. (Table 2; Figs 2c and 3).

Taken together, most of coronatine inducible proteins are important to plant photosynthesis, chloroplast organization, and positive regulation of defense. They were stable under normal conditions. When heat stress occurred, these proteins were unstable and degraded, while coronatine pretreatment increased their stability, and then it increased the stability of chloroplast ultrastructure and membrane to maintain a higher photosynthesis under heat stress.

### Coronatine decreased endogenous ABA production under heat stress

Some researches show that coronatine enters plant cells and causes stomata reopening, but the associated mechanisms of this action are still unclear. In our research, we focused on phytohormone ABA, which is closely related to stomata movement. Coronatine had no big difference on ABA production at the control temperature, while under heat stress, coronatine leaded to lower ABA production in both seedlings and flag leaves (Table 3). This result suggested that coronatine might be involved in stomata movement by regulating ABA levels. Thus, more CO_2_ entered plant cells, enhancing photosynthesis at some level. Acceleration of transpiration rate also cooled leaf temperature and facilitated uptake of water and nutrition, reducing the damage of high temperature. Thus, maintaining high relative leaf water content resulted from the balance of physiological process, not from stomata closure.

### Coronatine enhanced the capacity of photosynthesis to reduce yield loss under heat stress.

**Figure 4:**
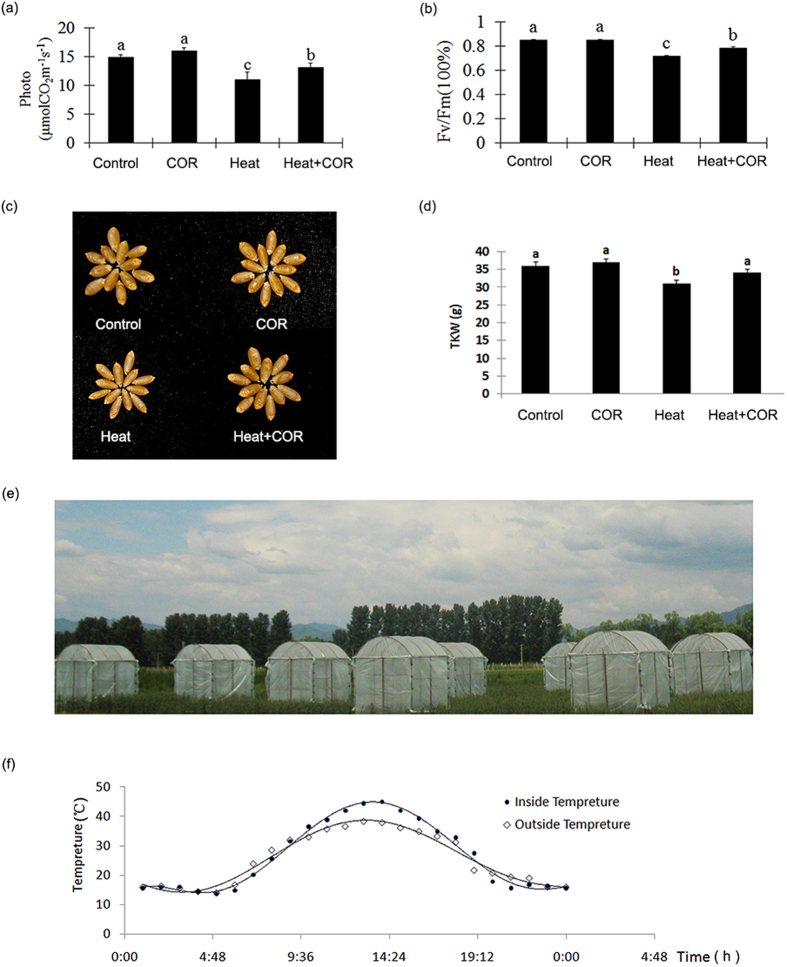
Coronatine induced photosynthesis and yield variation. (**a**) Photosynthetic performance of different treatment. Four treatments: Control: outside the shed for 7 days after water pretreatment for 24 hrs; COR: outside the shed for 7days after coronatine pretreatment for 24 hrs; Heat: inside the shed for 7 days after water pretreatment for 24 hrs; COR + Heat: inside the shed for 7 days after coronatine pretreatment no shed for 24 hrs. The photosynthetic capacity was shown as mean ± SD from 3 replicates (n = 20 lines/replicate; Bars labeled with different letter are significantly different at P > 0.05 as determined by LSD test). (**b**) Chlorophyll fluorescence of different treatment. The chlorophyll fluorescence was shown as mean ± SD from 3 replicates (n = 20 lines/replicate; Bars labeled with different letter were significantly different at P > 0.05 as determined by LSD test). (**c**) Phenotype of wheat grain in different treatment. (**d**) TKW (Thousand Kernel Weight) of different treatment corresponding with figure C. The TKW was shown as mean ± SD from 3 replicates (n = 20 lines/replicate; Bars labeled with different letter were significantly different at P > 0.05 as determined by LSD test). (**e**) Simplified shed for heat stress. The height of shed is 2.5 m, and 1.5 m width, 0.5 m height opened at the bottom for gas exchange. Picture was taken by Yuyi Zhou. (**f**) The different temperature between inside and outside the shed. The temperature recorded by auto-thermometer through the whole day.

We chose both photosynthesis and chlorophyll fluorescence kinetics to reveal the causative mechanism. When heat stress occurred, the photosynthetic rate of coronatine pretreated plants was 20.1% higher than it in control plants, and chlorophyll maximum quantum yield was 15.6% higher than the control (Fig. 4a,b). Mature grain was small and shriveled under heat stress, but coronatine pretreated kernels remained plump, with TKW (Thousand Kernel Weight) 8.2% higher than control (Fig. 4c,d). This result also revealed in field test (Zhou^33^, 2013, Fig. 4e,f). All result suggested that coronatine could reduce yield loss by enhancing photosynthetic performance under heat stress.

## Discussion

The structure of coronatine is similar to the phytohormone Jasmonates. Both have the COI1 plant receptor, so coronatine can manipulate plant hormone signaling^16^. During the last 20 years, the number of known plant hormones has grown from five to at least ten. The ubiquitin–proteasome pathway plays a central part in most hormone signaling pathways. The receptors of IAA, JA and Gibberellin are components of the SCF complex that consists of ASK, CUL and RBX^34^, so coronatine can affect the IAA, JA and Gibberellin pathways by the COI1 receptor. Coronatine also promotes stomata reopening through the E3 ligase subunit COI1. The pathway for stomatal closure involves triggering of the salicylic acid (SA) and abscisic acid (ABA) signaling pathways^20^,^35^. The mechanistic pathway of how coronatine regulates SA, ABA and NO to reopen stomata remains unclear; however, coronatine does affect these pathways. The precursor of the ethylene ACC is similar to CMA, which is the precursor of the coronatine. Some studies report that coronatine treatment can increase the key synthetase of ethylene 1-am inocyclopropane-1-carboxylate synthase, and improve plant production of ethylene^15^. In brief, coronatine can manipulate almost every hormone signaling pathway.

**Figure 5:**
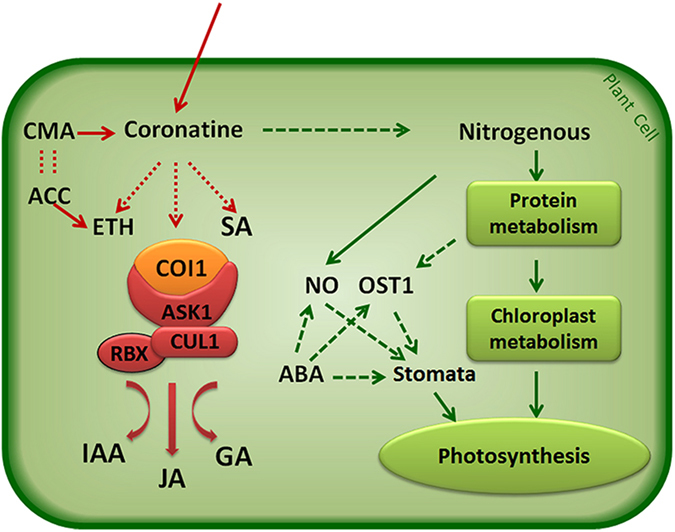
Model of coronatine worked in plant cell. When coronatine enters a plant cell, two branches of signaling occur. One is the hormone system. Coronatine and Jasmonate share the same receptor, COI1. The receptors of IAA, JA and Gibberellins are components of the SCF complex that consists of ASK, CUL and RBX, so coronatine can affect IAA, JA and Gibberellins pathways by receptor COI1. Coronatine also promotes stomata reopening through the E3 ligase subunit COI1. The pathway for stomatal closure involves triggering of the salicylic acid (SA) and abscisic acid (ABA) signaling pathways. The precursor of the ethylene ACC is similar to CMA, which is the precursor of coronatine. Thus coronatine improves plant production of ethylene. In brief, coronatine can manipulate almost every hormone signaling pathway. The other is physiological metabolism, coronatine mainly affects nitrogenous metabolism first. High concentration coronatine can lead to leaf chlorosis, but low concentration coronatine makes wheat leaves show a greener phenotype under heat stress. This function works by regulating protein processed and chloroplast ultrastructure to maintain photosynthetic performance in plants.

For our study, the plant phytotoxin coronatine showed a concentration effect similar to phytohormone auxin (IAA). High auxin amounts inhibit plant growth, while low auxin amounts can enhance plant growth. Similarly, high coronatine amounts lead to leaf chlorosis^22^, but low coronatine amounts could preserve the green phenotype of wheat leaves under heat stress. The phenotype of nitrogen loss caused us to focus on nitrogenous metabolism, since nitrogen is a key component of protein. Abiotic stresses usually cause protein dysfunction^35^. Maintaining proteins in their functional conformations is particularly important for cell survival under stress. Coronatine sensitive protein disulfide isomerase 2 is involved in protein structure rebuilding. CPN-60 alpha and some heat shock proteins are responsible for protein folding, assembly, translocation and degradation in many cellular processes and can assist in protein refolding under stress conditions. They play a crucial role in protecting plants against stress by reestablishing normal protein conformation. We found twenty two coronatine inducible proteins located in chloroplast, which is distributed in the chloroplast envelope, chloroplast stroma and chloroplast thylakoid membrane. Some proteins are a primary component of chloroplast ultrastructure. The 30S ribosomal protein S1 is involved in chlorophyll biosynthetic process. OsFTSH2 plays a key role in PSII associated light-harvesting complex II catabolic process. Coronatine pretreatment increased their stability, so it increased the stability of chloroplast ultrastructure and membrane to maintain a higher photosynthesis under heat stress. Take together, in physiological metabolism, coronatine mainly affects nitrogenous metabolism via regulating protein processes and chloroplast ultrastructure to maintain photosynthetic performance in wheat (Fig. 5).

## Methods

### Plant material, growth conditions, coronatine treatments and high temperature treatments.

Winter wheat (*Triticum aestivum L*.) cultivar Changwu134 was used in this study. It is a local cultivar in southwest China. Coronatine was prepared by the Centre for Crop Chemical Control, Department of Agronomy, China Agricultural University. Coronatine concentration was measured with high performance liquid chromatography (Milford, MA, USA). Experiments were carried out with seedlings cultured in a growth chamber characterized by 14/10 h photoperiod, 20 °C temperature, approximately 60% relative humidity and 400 μmol m^−2^ s^−1^ photon flux densities. At the three-leaved stage, seedlings were sprayed with 1 μmol/L coronatine. Twenty-four hours after treatment with coronatine, the concentration of internal coronatine was 4.18 ~ 9.83 ng/g in wheat leaves, some of the plants were subjected to heat stress induced by 40 °C (starting at 30 °C and then gradually increased 2 °C every 1 h and kept at 40 °C for 2 days), and control plants grow in 20 °C. Thus, treatments were: Control, COR, Heat, COR + heat. Each treatment was replicated three times and arranged into a completely randomized design. Two days after the heat treatment, plants were sampled and immediately preserved in liquid nitrogen. Field test was processed with simplified shed for heat stress. The height of shed is 2.5 m, and 1.5 m width, 0.5 m height opened at the bottom of shed for gas exchange. The temperature recorded by auto-thermometer through the whole day.

### Coronatine and ABA measurement in wheat leaves

After coronatine sprayed 24 h, collected seedlings and washed them with distilled water. For UPLC-TOF-MS analyses, 400 mg of fresh leaf tissue was grounded and extracted with 2 mL isopropanol. The crude extracts were dried under nitrogen at 40 °C, dissolved in 2 mL of MeOH/H_2_O (85:15, v/v) and passed through Sep-Pak Vac 3 mL C18 in order to extract chlorophyll and other interfering apolar compounds. To elute the compounds of interest from the cartridge, 1 mL of MeOH/H_2_O (85:15, v/v) was used. After drying under nitrogen, enriched extracts were dissolved in 200 uL MeOH/H_2_O (85:15, v/v) for UPLC-TOF-MS (Agilent LC/MS QQQ, Palo Alto, CA) analyses^22^,^36^.

### Photosynthetic rate, chlorophyll content and chlorophyll fluorescence

Photosynthetic rate (Pn), was measured with a portable photosynthesis system (LI-6400, LI-COR, Lincoln, USA). The concentrations of chlorophyll were determined using the SPAD-502 Chlorophyll Meter Model (Ectck, China). Chlorophyll fluorescence was measured by the PAM-2000 chlorophyll fluorescence system (Heinz Walz, Effieltrich, Germany). After a dark adaptation period of 30 min, minimum fluorescence (Fo) was determined by a weak red light (0.06 mmol m^−2^ s^−1^), and maximum fluorescence of dark adapted leaf (Fm) was measured during a subsequent saturated light pulse (3000 mmol m^−2^ s^−1^ for 0.8 s). The steady state fluorescence (Fs) reaching within 7–8 min was thereafter recorded and a second saturating pulse of white light (3000 mmol m^−2^ s^−1^) was imposed to determine the maximum fluorescence level in the light-adapted state (Fm). Fv/Fm reflects the potential maximum photosynthetic capacity of a plant, Fv/Fm = (Fm – Fo)/Fm.

### Protein extraction

1 g of wheat leaves was randomly collected and finely ground in liquid nitrogen. Protein extraction was performed according to Rinalducci *et al.* (2011)^37^ with some modifications. Three biological replicates were used. The powder was suspended (1 g/mL) in chilled lysis buffer in acetone containing 0.007% DTT and 1% plant protease inhibitor cocktail (Bio-Rad, USA). The mixture was incubated at −20 °C for at least 1 h, and then centrifuged at 12000 r for 15 minutes before the supernatant was collected.

### Two-dimensional electrophoresis

IEF was performed using the Bio-Rad-Protean-IEF-Cell-System. Twenty-four centimeter IPG strips (Bio-Rad, USA) pH 4–7 were passively rehydrated overnight with 750 μg of protein in 300 μl of solution containing 1% carrier ampholyte (Bio-lyte 4–7; Bio-Rad, USA). The total product time × voltage applied was 63,500 Vh for each strip at 20 °C. Strips were subsequently reduced (1%DTT, 15 min) and alkylated (2.5% IAA, 15 min) during the equilibration step (30 min in 50 mM Tris–HCl pH8.8, 6 M urea, 30% glycerol v/v, 1%SDS, bromophenol blue). Equilibrated strips were then placed on SDS-polyacrylamide gels, 22 cm × 26 cm, 15% acrylamide, and sealed with 0.5% agarose. Protein spots were stained by coomassie blue. To ensure protein pattern reproducibility, three technical replicates were done.

### Image analysis

Two dimension gel images were digitized using a flatbed scanner (model Image Scanner-II, GE Healthcare) with a resolution of 300 dpi and 16-bit greyscale pixel depth. Image analysis was carried out with Image Master 7.0, which allows spot detection, background subtraction, and protein spot OD intensity quantification. Spot quantity values were normalized in each gel dividing the raw quantity of each spot by the total quantity of all the spots included in the standard gel. For each protein spot, the average spot quantity value and its variance coefficient in each group was determined. The least significant difference (LSD) test was used to determine significant differences among group means. Protein spots were cut out from coomassie blue stained gels and subjected to trypsin digestion according to Shevchenko^38^ with minor modifications. Peptide mixtures were separated using Auto-flex2 (Germany, Brook) system. A sample volume of 1 μL was loaded by the autosampler. Gene function and location analysis were downloaded from the NCBI and TAIR website. Each protein was classified with respect to its cellular component, biological process, and molecular function. Protein expression pattern analysis was performed using Cluster 3.0. The data were analyzed statistically according to a randomized block design using SAS statistical software. The least significant difference (LSD) was calculated for the significant data at P ≤ 0.05.

### RNA extraction, cDNA synthesis and qRT-PCR

Corresponding genes were double confirmed by quantitative RT-PCR. Total RNAs were prepared from wheat leaves treated with DNase before being subjected to cDNA synthesis using Superscript III reverse transcriptase primed by oligo dT. Quantitative RT-PCR was performed in 96-well plates with an ABI 7500Fast real-time PCR system using the SYBR Green II mix (Applied Biosystems). PCR primers were designed using the Primer Express Software DNAman. Three biological replicates were performed, and the reactions were performed in triplicate for each run. The comparative CT method was used to evaluate the relative quantities of each amplified product in the samples. The threshold cycle (CT) was automatically determined for each reaction by the system according to the default parameters^39^. The specificity of the PCR was determined by dissociation curve analysis of the amplified products using the standard method installed in the system^26^. Primers used for qRT-PCR is listed in Table 4.

### Protoplast preparation

Protoplast isolation was based on the protocol for wheat mesophyll protoplasts provided online by J.Sheen’s laboratory http://genetics.mgh.harvard.edu/sheenweb/ with some modifications. Coronatine was added in the solution directly.

## Additional Information

**How to cite this article**: Zhou, Y. *et al.* Phytotoxin coronatine enhances heat tolerance via maintaining photosynthetic performance in wheat based on Electrophoresis and TOF-MS analysis. *Sci. Rep.* **5**, 13870; doi: 10.1038/srep13870 (2015).

## Figures and Tables

**Table 1:** The effect of wheat root induced by heat stress and COR.

**Treatment**	**Length (cm)**		**SurfArea (cm**^**2**^)		**AvgDiam (mm)**		**RootVolume (cm**^**3**^)	
Control	331	d	33.5	c	0.324	b	0.270	d
COR	344	c	35.5	c	0.328	a	0.291	c
Heat	409	b	40.9	b	0.319	c	0.327	b
Heat + COR	445	a	45.8	a	0.321	c	0.376	a

Each value represents the mean ± SD (n = 10). Bars showing the same latter are not significantly different at P ≤ 0.05 as determined by LSD test.

**Table 2:** List of coronatine inducible proteins identified by MODI-TOF-TOF.

**NO**	**Name in wheat**	**NCBI GI No.**	**Homologue At Arab.**	**Mass**	**Mascot score (>72)**	**pI**	**No. of peptides**	**Location in plant**	**Function in plant**
1	protein disulfide isomerase 2 precursor	gi|13925726	AT1G21750	56691	139	5.03	19	Chloroplast, endoplasmic reticulum, membrane, cell wall, vacuole.	Embryo development, metabolic process, cell death, response to salt stress, response to zinc ion.
2	predicted protein	gi|326499830	AT4G09650	26696	85	4.78	28	Chloroplast, chloroplast envelope, membrane, plastoglobule, thylakoid.	Negative regulation of defense response, defense response to bacterium, ion transmembrane transport, proton transport, response to cold, cytokinin, salicylic acid biosynthetic process.
3	cp31BHv	gi|226533870	AT4G24770	18993	96	4.85	23	Chloroplast, thylakoid.	Cold acclimation, RNA modification, RNA processing, innate immune response.
4	predicted protein	gi|326508923	AT5G09650	31829	86	5.46	22	Chloroplast, membrane, thylakoid.	Defense response to bacterium, metabolic process, response to cadmium ion, response to salt stress.
5	plastid glutamine synthetase isoform GS2	gi|71362640	AT5G35630	47016	178	5.75	21	Apoplast, chloroplast, cytosolic ribosome, membrane, mitochondrion, thylakoid.	Aging, ammonia assimilation cycle, metabolic process, response to cadmium ion, response to salt stress.
6	predicted protein	gi|326487794	AT3G63490	37619	123	8.25	25	Chloroplast, membrane, nucleus, ribosome.	Chloroplast relocation, ncRNA metabolic process, pentose-phosphate shunt, photosynthesis, protein targeting to chloroplast, thylakoid membrane organization, transcription from plastid promoter, translation.
7	OsFTSH2	gi|75114857	AT2G30950	72607	112	5.54	33	Chloroplast, membrane, thylakoid.	ATP catabolic process, PSII associated light-harvesting complex II catabolic process, metabolic process, photoinhibition, reactive oxygen species metabolic process, thylakoid membrane organization.
8	pathogenesis-related protein 4	gi|49615737	AT3G04720	15547	94	4.75	15	Endomembrane system	Killing of cells of other organism, response to nitrate, defense response to fungus, incompatible interaction, response to ethylene stimulus, response to herbivore, response to salt stress, response to virus, systemic acquired resistance.
9	predicted protein	gi|326504766	AT3G46230	17533	76	5.54	47	Cytoplasm	Response to heat.
10	hypothetical protein	gi|225467488	AT1G05380	68465	76	6.08	31	Nucleus	Regulation of transcription, DNA-dependent.
11	phosphoribulokinase	gi|125580	AT1G32060	45406	106	5.84	41	Apoplast, chloroplast, membrane, thylakoid.	Biosynthetic process, defense response to bacterium, phosphorylation, response to cold.
12	30S ribosomal protein S1	gi|149391139	AT5G30510	23215	78	5.03	32	Chloroplast, thylakoid.	Chlorophyll biosynthetic process, photosynthesis, response to cytokinin stimulus, thylakoid membrane organization.
13	cysteine synthase	gi|585032	AT4G14880	34207	188	5.48	19	Apoplast, chloroplast, cytosol, membrane, nucleus, peroxisome, plasma membrane, vacuolar membrane.	Aging, cysteine biosynthetic process, response to cadmium ion, response to salt stress, response to temperature stimulus, response to wounding, water transport.
14	protein disulfide isomerase 2 precursor	gi|13925726	At1g21750	56691	162	5.03	18	Chloroplast, endoplasmic reticulum, membrane, cell wall, protein storage vacuole, thylakoid, vacuole.	Embryo development, cell death, response to cytokinin stimulus, response to endoplasmic reticulum stress, response to salt stress, response to zinc ion, systemic acquired resistance.
15	predicted protein	gi|326496613	AT2G21170	32679	93	7.04	26	Apoplast, chloroplast. cytosol, mitochondrion, thylakoid.	Chloroplast organization, primary root development, reductive pentose-phosphate cycle, triglyceride mobilization.
16	predicted protein	gi|326523691	AT3G52150	26261	74	9.07	29	Chloroplast, thylakoid.	Isopentenyl diphosphate biosynthetic process, mevalonate-independent pathway, photosynthetic, protein targeting to chloroplast.
17	Thiol-specific antioxidant protein	gi|326496957	AT3G11630	23398	118	5.48	9	Apoplast, chloroplast, thylakoid.	MAPK cascade, defense response to bacterium, oxidation-reduction process, response to cold, regulation of plant-type hypersensitive response, response to chitin, response to cold, salicylic acid biosynthetic process.
18	unnamed protein product	gi|755762	AT5G35630	46902	96	5.75	18	Apoplast, chloroplast, cytosolic ribosome, membrane, mitochondrion, thylakoid.	Aging, ammonia assimilation cycle, metabolic process, response to cadmium ion, response to salt stress.
19	S-like RNase	gi|20271131	AT1G26820	28320	120	6.03	20	Endomembrane system	Aging, metabolic process.
20	chloroplast fructose-bisphosphate	gi|223018643	AT4G38970	42217	103	5.94	19	Apoplast, chloroplast, plastoglobule, thylakoid.	Metabolic process, pentose-phosphate shunt, response to abscisic acid stimulus, response to cadmium ion.
21	CCMP1545	gi|303290985	AT2G31400	90159	73	9.53	27	Chloroplast	Chloroplast-nucleus signaling pathway, mitochondria-nucleus signaling pathway.
22	ps16protein	gi|2443390	AT4G24770	31829	80	4.55	31	Chloroplast, thylakoid.	Required for editing and stability of specific chloroplast mRNAs, RNA modification, and RNA processing, innate immune response.
23	Chl sHSP	gi|156711774	AT4G27670	13135	74	5.09	5	Chloroplast	Response to heat, response to high light intensity, response to hydrogen peroxide.
24	CPN-60 alpha	gi|134102	AT2G28000	57656	240	4.83	37	Apoplast, chloroplast, cytosolic ribosome, membrane, mitochondrion, thylakoid.	Chloroplast organization, embryo development, protein folding.
25	70 HSP	gi|147860809	AT4G24280	74472	98	5.01	37	Chloroplast, mitochondrion, nucleus, plastid stroma, thylakoid.	Protein folding, protein targeting to chloroplast, response to cadmium ion, response to cold.
26	GST	gi|20067415	AT1G78380	25098	159	6.35	54	Chloroplast, cytoplasm, cytosol, plasma membrane, vacuolar membrane.	Cellular response to water deprivation, metabolic process, response to cadmium ion, response to oxidative stress, toxin catabolic process.
27	peroxidase 4	gi|57635153	AT5G05340	33438	88	5.78	24	Apoplast, cell wall, cytosol.	Oxidation-reduction process, response to oxidative stress.

Protein name and GI number are from NCBI BLAST search of PSBP_WHEAT. Theoretical pI was calculated on the Matrix Science web site (http://www.matrixscience.com). TAIR accession number is the closest homologue in Arabidopsis thaliana. Location and function analysis are searched from NCBI and TAIR website.

**Table 3:** ABA production in wheat seedlings and flag leaves.

**Treatment**	**Seedlings ABA (ug·g**^**−1**^)	**Flag leaves ABA (ug·g**^**−1**^)
Control	0.542a	0.506b
COR	0.545a	0.493b
Heat	0.536b	0.533a
Heat + COR	0.454c	0.459c

Each value represents the mean ± SD (n = 10). Bars showing the same latter are not significantly different at P ≤ 0.05 as determined by LSD test.

**Table 4:** Primers used for qRT-PCR.

**Gene**	**Primers**
*actin*	5′GGAATCCATGAGACCTAC3′5′GACCCAGACAACTCGCAAC3′
*cp31BHv*	5′ATCAGCGGAAGGCTTCTGAACGTA3′5′CTCGCTGAACAATTGCACCAACCT3′
*Peroxidase 4*	5′GCAACGGCCATTATAGCGCTTTCT3′5′TTTGCACAGGGCCATTGACATACG3′
*Chl sHSP*	5′CCCATGGGAGATCAAAGAAGGTGA3′5′AACAAGCATCTTCTCCTCCACCCA3′
*GST*	5′TTACCCACAGAACAAGGTGC3′5′CCATCACTGAACTTTCCCAGG3′
*30S ribosomal protein S1*	5′TTGGGAACTGTCGAGAGCCTGAAA3′5′AGAACTGTCGAGATGTCCGCAACA3′
*CCMP1545*	5′CCAGGATAACGGCATGGTGAACAA3′5′AGTGTGATGATGGATGAGAACGCG3′
*70 HSP*	5′CCAGGATAACGGCATGGTGAACAA3′5′ACGGGAACCAGTGTGATGATGGAT3′
*CPN-60 alpha*	5′GTGCAGCTCTGATTCGTGAG3′5′ATGCCCAGCTTGATGATTTC3′
*Ps16protein*	5′TGCTGCGTAAGAGAGACACGAT3′5′TGAGAACGTGGAAGCAACAG3′
